# An Uncommon Cause of Gastrointestinal Bleeding in an 84-Year-Old Female

**DOI:** 10.1155/2013/940271

**Published:** 2013-05-12

**Authors:** Suartcha Prueksaritanond, Aram Barbaryan, Alaa M. Ali, Aibek E. Mirrakhimov

**Affiliations:** Saint Joseph Hospital/University of Illinois at Chicago, Department of Internal Medicine, 2900 N. Lake Shore, Chicago, IL 60657, USA

## Abstract

The estimated annual incidence for drug-induced thrombocytopenia is 10 per million. Although fatal consequences are uncommon, life-threatening hemorrhage can occur due to spontaneous bleeding. We report a case of 84-year-old Caucasian female who presented to the emergency department with multiple episodes of bloody bowel movements. One week prior to this admission, she was started on trimethoprim-sulfamethoxazole for the treatment of skin abscess. On admission laboratory results showed platelet count of 4 × 10^3^/mm^3^ and hemoglobin of 10.2 g/dL. Because of unstable vital signs, the patient was transferred to the intensive care unit where she received multiple units of platelet and blood transfusion. Drug-induced thrombocytopenia due to TMP/SMX was suspected. Intravenous methylprednisolone was started as well as immune globulin with good clinical response.

## 1. Introduction

Since trimethoprim-sulfamethoxazole (TMP/SMX) has been available in 1968, it has been used for the treatment of various infections, including bacterial and nonbacterial infections [1]. Its hematologic adverse effect, particularly thrombocytopenia, is well documented [2–4]. The overall estimated incidence for drug-induced thrombocytopenia (DITP) is 10 per million populations per year [5]. Although fatal consequences are uncommon, life-threatening hemorrhage can occur when platelets are less than 10 × 10^3^/mm^3^ due to spontaneous bleeding. We present an uncommon case of spontaneous life-threatening hemorrhage due to severe thrombocytopenia induced by TMP/SMX.

## 2. Case Presentation

An 84-year-old Caucasian female presented to the emergency department with multiple episodes of bloody bowel movements developed acutely on the day of admission. Her symptoms were associated with dizziness and lightheadedness. Patient denied any other symptoms including fever, nausea, abdominal pain, or prior history of gastrointestinal bleed. 

Her medical history is relevant for hypertension, hyperlipidemia, osteoarthritis, and non-Hodgkin lymphoma, in remission for the last 8 years. Surgical history was positive for mitral valve repair at the age of 67 and bilateral total hip arthroplasty at the age of 74 years. Her medications included metoprolol, rosuvastatin, digoxin, and low-dose aspirin. She also reported taking ibuprofen occasionally for osteoarthritis. She denied taking other anticoagulants. Despite her age, the patient never had a screening colonoscopy. She had history of sulfa allergy but did not remember the reaction. 

One week prior to this admission, she was started on TMP/SMX for an abscess on her right hip. She took the antibiotic for 2 days and discontinued it 5 days prior to presentation after remembering history of sulfa allergy. Blood work prior to starting antibiotic revealed normal platelets and hemoglobin values. On presentation, she was found to be pale and diaphoretic. Vital signs were temperature 36.3°C, pulse 83/min, respiratory rate 18/min, and blood pressure 57/38 mmHg. The examination revealed wet purpura on the right buccal mucosa and scattered petechial lesions on both lower extremities. Laboratory results showed platelet count of 4 × 10^3^ (reference range 140–400 × 10^3^/mm^3^), hemoglobin of 10.2 (reference range 13.5–17.0 g/dL), and white blood count of 8.4 × 10^3^ (reference range 4.2–11.0 × 10^3^/mm^3^). Comprehensive metabolic profile, coagulation studies, lactate dehydrogenase, and lactic acid levels were within normal limit. The peripheral blood smear showed markedly decreased platelet count with no schistocytes. The differential counts for white blood cells were normal. All cultures were obtained and broad-spectrum antibiotics with vancomycin and piperacillin/tazobactam were started. The patient initially received fluid resuscitation with crystalloid while in emergency department but failed to raise her blood pressure. Due to persistent hypotension, she was admitted to the intensive care unit (ICU). During the first day in ICU, she continued to pass bloody bowel movements. The patient was aggressively resuscitated necessitating the use of vasopressors. Two units of platelets and two units of packed red blood cells were initially transfused. However, they failed to raise her platelets above 10 × 10^3^/mm^3^ and her hemoglobin declined. Drug-induced thrombocytopenia due to TMP/SMX was suspected and intravenous methylprednisolone was started as well as immune globulin. While she continually being transfused, bleeding scan was done on hospital day 2 and revealed gastrointestinal hemorrhage in the area of hepatic flexure. The bleeding finally stopped when platelet levels reached >50 × 10^3^/mm^3^ on hospital day 3. Overall, 7 units of packed red blood cells, 8 units of platelets, and 2 units of fresh frozen plasma were transfused during the first 3 days of admission. Despite high suspicion for drug-induced thrombocytopenia, platelet antibodies were undetectable, although the sample was sent on hospital day 2, after systemic corticosteroid and immune globulin had begun. Also, the test was ordered as indirect platelet antibody. Colonoscopy on hospital day 3 showed a small oozing arteriovenous malformation at cecum which was cauterized and clipped. All cultures were negative except for wound culture which revealed methicillin-sensitive *Staphylococcus epidermidis*. Antibiotics were later switched to cefazolin. Nine days after discontinuation of TMP/SMX, her platelet count increased to 104 × 10^3^/mm^3^ and her hemoglobin remained stable at 11 g/dL. Systemic corticosteroid was given for the total of 5 days. She was discharged on hospital day 7. Upon discharge, her platelet count was 207 × 10^3^/mm^3^ and hemoglobin was 13.1 g/dL.  Table 1 showed trending of complete blood count during hospitalization. 

## 3. Discussion

TMP/SMX has long been associated with thrombocytopenia but its exact mechanism is not yet established. Immune-mediated reactions including production of drug-dependent antibodies and drug-induced autoantibodies have been implicated in platelet destruction [5]. Although overall incidence of DITP is low, the incidence of TMP/SMX-induced thrombocytopenia was reported to be as high as 38 per million users per week [5]. Nevertheless, most cases resolved spontaneously without treatment. Despite its safety profile, fatal hemorrhages can occur when platelet is less than 10 × 10^3^/mm^3^. Based on our MEDLINE search, two cases have been reported to associate spontaneous hemorrhage with severe thrombocytopenia due to TMP/SMX.

The diagnosis of DITP required strong suspicion. The condition should be suspected in any patient who presents with acute thrombocytopenia with uncertain etiology. Presence of severe thrombocytopenia (<20,000/mm^3^) increases the likelihood of DITP [5]. Furthermore, an exposure time of 5–7 days is usually needed to produce sensitization in a first-time user [5]. Clinical criteria defined by George and Aster can also be used to evaluate the suspected agent [6]. Demonstration of drug-dependent antibodies (DDAb) can help confirm the etiology as well. The assay performs by testing patient's serum or plasma against normal platelets in the presence and the absence of the suspected drug [7]. Proper negative control also performs with normal serum to ensure the drug does not nonspecifically cause positive result [7]. The result is positive when the patient's sample interacts with normal platelets in the presence of the drug only [7]. However, such testing is not readily available and requires considerable amount of time; hence, it is not suitable for acute patient care where urgent intervention may be required [5, 6]. Moreover, these tests can produce negative results. Reasons for discrepancies include low sensitivity of available assay to detect antibodies, poor solubility of the drugs in aqueous medium which makes it difficult to incorporate into in vitro assays, and a metabolite rather than the primary drug which may be responsible for the thrombocytopenia [6, 8]. Once DITP is established, the patient may be sensitized to that particular agent indefinitely [5].

Thrombocytopenia usually resolves spontaneously within 1-2 days once the drug is discontinued based on its half-life [6, 9]. However, platelet transfusions may be needed where the bleeding is a concern, especially in the presence of severe thrombocytopenia and “wet purpura” [9]. Systemic corticosteroids are often given due to suspected immune-mediated process and reported to be beneficial [9], however, only through series of case reports. Currently, there are no large studies to support their efficacy. Intravenous immune globulin and plasma exchange have been used in acutely ill patients as well, but the benefit is also uncertain [8]. Despite the uncertainty of the treatment, their benefits may outweigh the risk when facing life-threatening situation. 

In our patient, DITP seems to be the most likely cause based on history of severe acute thrombocytopenia without evidences of other precipitating causes. On further workup, there is no evidence of severe systemic infection, disseminated intravascular coagulation (DIC), hemolytic uremic syndrome (HUS), or thrombotic thrombocytopenic purpura (TTP). There was no history of anticoagulant use and her coagulation tests were normal. Since the only medication identified prior to acute episode of thrombocytopenia was TMP/SMX and the blood work done prior to starting the medication was normal, it is likely that TMP/SMX was the causative agent. Furthermore, our patient has been preexposed to TMP/SMX; reintroduction of the drug could only magnify the antibody response. Ibuprofen, another medication taken by the patient, is also associated with thrombocytopenia. However, the suspicion is low due to history of chronic use and no prior reports of abnormal blood work. The negative results on platelet antibodies can also be explained by several reasons: (1) the assay that was used was not specific to DDAb and less sensitive [7] and (2) there was a delay in sample collection. Although having positive DDAb is helpful in diagnosing DITP, the results can be equivocal [7]. Nonetheless, it is unclear if the rise of platelet count was the response to immune globulin and systemic steroid. Retesting of the medication to confirm the causative agent was not considered in this case due to the seriousness of her conditions associated with DITP.

In summary, TMP/SMX is a commonly used antibiotic which has been associated with DITP. Although most patients recovered spontaneously without treatment, fatal hemorrhage may occur in presence of severe thrombocytopenia. Once the offending agent has been identified and promptly discontinued, patients should be advised to avoid the medication permanently. Furthermore, routine monitoring of complete blood count is uncommon in short-term use of TMP/SMX. Prescribing physicians and patients should be aware of its adverse effect and patient should be instructed to promptly seek medical attention when needed.

## Figures and Tables

**Table 1:** Complete blood counts during hospitalization.

Complete blood count (CBC)									
Hospital day	1		2		3	4	5	6	7
WBC* (×10^3^/mm^3^)	8.4		6.4	10.5	7.9	7.0	7.7	7.1	8.1
Hb* (g/dL)	10.2	8.4	7.5	8.4	10.4	11.0	11.2	11.0	13.1
Platelet (×10^3^/mm^3^)	4	10	3	16	54	104	139	156	207

*WBC: white blood cell count. Hb: hemoglobin.
